# Estimating the height of elderly nursing home residents: Which equation to use?

**DOI:** 10.1371/journal.pone.0205642

**Published:** 2018-10-23

**Authors:** Marcos Felipe Silva de Lima, Larissa Praça de Oliveira, Natália Louise de Araújo Cabral, Laura Camila Pereira Liberalino, Ursula Viana Bagni, Kenio Costa de Lima, Clélia de Oliveira Lyra

**Affiliations:** 1 Postgraduate of Public Health, Federal University of Rio Grande do Norte, Natal, Brazil; 2 Department of Nutrition, Federal University of Rio Grande do Norte, Natal, Brazil; 3 Department of Dentistry, Federal University of Rio Grande do Norte, Natal, Brazil; University of Rhode Island, UNITED STATES

## Abstract

Estimating equations can be used when measuring the height of elderly persons is not possible. However, such methods are not always applicable; therefore, it is necessary to consider several aspects, such as sex, age, and ethnicity of the studied population to generate these equations. This study aimed to compare and validate methods of estimating the height of the Brazilian elderly nursing home residents. An accuracy study was conducted with 168 elderly persons. A total of 23 equations were quantitatively evaluated by plotting the differences in means, the Student’s t-test for paired samples, the coefficient of determination (R^2^), the root-mean-square error (RMSE), the interclass correlation coefficient (ICC), and by graphic analysis of the residuals. A significance value of *p* <0.05 was adopted. An equation was considered applicable when it had R^2^ >0.7, the lowest RMSE among the equations evaluated, ICC >0.7, and a confidence interval of 95%, with the smallest difference between the upper and lower limits. A greater mean height was noted among younger elderly persons and elderly men compared to up to 80 years and women elderly. Quantitative analysis revealed that equation for Puerto Ricans, using knee height and age, was the most applicable for the overall population (ICC = 0.802). The same equation was applicable for the elderly Brazilian male participants (ICC = 0.838) and for those aged 60–69 years (ICC = 0.895). None of the equations used were applicable for the height estimation of elderly women or individuals aged 70 years or more.

## Introduction

The aging process increases physical and functional decline, and when combined with institutionalization, it can compromise autonomy and independence[1]. Nutritional status can be affected by physiological alterations and the diseases associated with aging and result in deficits or excesses in indicators[2]. Therefore, the evaluation of nutritional status plays an important role when monitoring situations of nutritional risk.

Body height is a measure which represents, in addition to genetic factors, socioeconomic, demographic, and health conditions, as well as the conditions of the physical and social environments a person has experienced. In this sense, linear growth is related to environmental development and the health conditions to which the individual is exposed[3–5]. Height declines, from the age of 40 onwards, by about 1.0–2.5 cm per decade, and this decline becomes more pronounced at advanced ages. These changes are the result of the morphological changes that occur during the aging process, such as the reduction of the plantar arch, the increased curvature of the spine, and the flattening of the intervertebral discs [6,7].

The measurement of height is also required to calculate the body mass index (BMI), the most commonly used indicator in the anthropometric assessment of the elderly. Despite the importance of this measure in monitoring nutritional status and management, measurement of height is often impossible for elderly who are bedridden and difficult for elderly patients with restricted mobility. In such cases, height may be estimated based on other measurements of lengths and body segments. The main methods cited in scientific literature are the protocols and equations for the prediction of height developed by Chumlea et al.[8]. These equations were developed for Caucasian American elderly persons, aged 65–90 years and from only one state of the USA. Other equations have been developed by the same group of authors seeking greater representation of the American population, encompassing those of African descent and Mexican origin[9,10]. Latin American and Brazilian authors have developed equations for the estimate of height of these people [11–18] The application of these equations must consider the compatibility of the characteristics of the population that gave rise to the equations and which is to be assessed.

The applicability of simplified methods for the early detection of possible changes in anthropometric nutritional status among the elderly nursing home residents is important because this people are often fragile. In addition, prediction equations are necessary for dietary interventions in older age, particularly in those who cannot have their height (as part of BMI measure) directly measured. This study aimed to compare and validate height estimation methods in elderly Brazilian living in nursing homes. It is believed that the study can contribute to the monitoring of anthropometric nutritional status for future nutritional interventions in this population and support recommendations for equations that estimate height.

## Materials and methods

A cross-sectional, census-based study of elderly residents of nursing homes in Natal city, in the state of Rio Grande do Norte, a city in northeastern Brazil, was carried out between October 2013 and June 2014. The study population consisted of all elderly nursing home residents in this period. The inclusion criteria were nursing home residents aged over 60 years who were present at the time of data collection. The exclusion criteria were individuals confined to bed, for whom it was impossible to properly measure height or who had a physical disability that prevented the taking of anthropometric measurements, such as patients who underwent orthopedic surgery.

The anthropometric evaluation was performed by two trained anthropometrists. A calibrated equipment was used for evaluation, and the technical error measurement (TEM) was identified between the evaluators. The purpose of interevaluator TEM is to assess the agreement between the evaluators to ensure that, regardless of who is carrying out the measures, the result would be the same. The evaluators were considered qualified to perform the anthropometric measurements, according to the classification of experienced anthropometrists proposed by Pederson and Gore[19].

The following anthropometric measurements were performed: height and knee height according to Lohman et al. [20], ulna length and demi-span according MAG [13], and half arm span according to Kwok and Whitelaw [14]. All the measures were evaluated in duplicate. Height was measured using a portable stadiometer, with a non-slip base, with a measuring range from 0 to 2200 mm and a 1.0-mm precision. Knee height was taken using an anthropometer, with a measuring range from 0 to 1000 mm and a 1.0-mm precision. Ulna length, half arm span, and demi-span were measured using a segment caliper, with a measuring range from 0 to 2800 mm and a 1.0-mm precision.

Measured height was used as a standard measurement for comparing the height estimation equations. The estimated height was calculated from the anthropometric measurements and age, using the equations shown in Table 1.

**Table 1:** Methods of height estimates analyzed in the present study.

Women		Men
**Chumlea et al.**[8]		
**Eq. 1**	H (cm) = 84,88 + (1,83 x KH)–(0,24 x AGE)	H (cm) = 64,19 + (2,02 x KH)–(0,04 x AGE)
**Chumlea; Guo**[9] **(2 –white; 3 –black)**		
**Eq. 2**	H (cm) = 75 + (1,91 x KH)–(0,17 x AGE)	H (cm) = 59,01 + (2,08 x KH)
**Eq. 3**	H (cm) = 58,72 + (1,96 x KH)	H (cm) = 95,79 + (1,37 x KH)
**Chumlea et al.**[10] **(4 –white; 5 –black; 6 –mexican)**		
**Eq. 4**	H (cm) = 82,21 + (1,85 x KH)–(0,21 x AGE)	H (cm) = 78,31 + (1,94 x KH)–(0,14 x AGE)
**Eq. 5**	H (cm) = 89,58 + (1,61 x KH)–(0,17 x AGE)	H (cm) = 79,69 + ((1,85 x KH)–(0,14 x AGE)
**Eq. 6**	H (cm) = 84,25 + (1,82 x KH)–(0,26 x AGE)	H (cm) = 82,77 + (1,83 x KH)–(0,16 x AGE)
**Bermúdez; Tucker**[11] **(7–8 –hispanic; 9–10 –puerto rican)**		
**Eq. 7**	H (cm) = 59,29 + (1,92 x KH)	H (cm) = 70,28 + (1,81 x KH)
**Eq. 8**	H (cm) = 68,68 + (1,90 x KH)—(0,123 x AGE)	H (cm) = 76,02 + (1,79 x KH)—(0,07 x AGE)
**Eq. 9**	H (cm) = 55,98 + (1,99 x KH)	H (cm) = 53,42 + (2,13 x KH)
**Eq. 10**	H (cm) = 66,80 + (1,94 x KH)—(0,123 x AGE)	H (cm) = 52,95 + (2,13 x KH)—(0,006 x AGE)
**Palloni; Guend**[12]		
**Eq. 11**	H (cm) = 94,0667 + (1,2110 x KH)	H (cm) = 98,1691 + (1,2948 x KH)
**Eq. 12**	H (cm) = 106,0251 + (1,1914 x KH)—(0,1539 x AGE)	H (cm) = 105,9638 + (1,2867 x KH)—(0,1030 x AGE)
**Malnutrition Advisory Group (MAG)**[13]		
**Eq. 13**	Ulna length^a^	
**Eq. 14**	Knee height^a^	
**Eq. 15**	Demi span^a^	
**Kwok; Whitelow**[14]		
**Eq. 16**	H (cm) = 2 x Half arm span	
**WHO**[15]		
**Eq. 17**	H (cm) = (0,73 x (2 x Half arm span) + 0,43)	
**Lera et al.**[16] **(18 –brazilian; 19 –chilean; 20 –mexican)**		
**Eq. 18**	H (cm) = (1,85 x KH)—(0,11 x AGE) + 69,87	H (cm) = (1,96 x KH)—(0,08 x AGE) + 67,2
**Eq. 19**	H (cm) = (1,78 x KH)—(0,10 x AGE) + 75,17	H (cm) = (2,09 x KH)—(0,10 x AGE) + 64,88
**Eq. 20**	H (cm) = (1,99 x KH)—(0,06 x AGE) + 63,88	H (cm) = (1,99 x KH)—(0,06 x AGE) + 63,88
**Silveira et al.**[17]		
**Eq. 21**	H (cm) = 51,875 + (2,184*KH)	H (cm) = 72,803 + (1,830*KH)
**Rabito et al.**[18]		
**Eq. 22**	H (cm) = 58,045 - (2,965 x Sex)—(0,07309 x AGE) + (0,5999 x ulna length) + (1,094 x Half arm span)	
**Eq. 23**	H (cm) = 63,525 - (3,237 x Sex)—(0,06904 x AGE) + (1,293 x Half arm span)	

^a^: standardized table available in < https://www.bapen.org.uk/pdfs/must/must_explan.pdf >

Data were analyzed using the IBM SPSS software version 14.0 for Microsoft Windows. The estimating equations were analyzed by qualitative and quantitative methods. Qualitatively analysis does not require a complex model, since the methods of stature estimation are standardized. Nearly all estimates used knee height or the binomial “knee height + age.” A few other measures were used, such as the ulna length, half arm span, and demi-span. Therefore, it was performed from the plausibility and ease of anthropometric measurement that makes up the estimating equation, considering the positioning that the evaluated elderly should be submitted for the correct anthropometric measurement. The entire population was assessed quantitively and stratified by sex (male and female) and age (60–69 years, 70–79 years, and 80 years or over).

For the preparation of the database for the multivariate analysis, an analysis was conducted regarding the missing values and multivariate outliers. In the analysis of missing values, non-random errors were verified, and the missing values were obtained by linear interpolation with data stratified by sex. Regarding the multivariate detection of atypical observations, the distance D2 of Mahalanobis was measured and assumed as a multivariate outlier when the D2 value divided by the number of variables analyzed was greater than or equal to 3.0. Thus, six individuals representing multivariate outliers were excluded from the data analysis.

To characterize the study population, the anthropometric measures were evaluated, and Student’s t-tests for independent samples tests were performed to compare the average values between the sexes. Analysis of variance was performed to compare the means among the age groups studied. Bonferroni post-hoc was performed. The measured and estimated height were evaluated using descriptive analysis, the Student’s t-test for repeated measures, and plotting of the error bars of the mean difference and the corresponding 95% confidence interval (CI). Concordance analysis was performed using the coefficient of determination (R^2^), the root-mean-square error (RMSE), the intraclass correlation coefficient (ICC), and their corresponding CIs (95%). The assumptions of linear regression analysis were tested by Bland-Altman plot, and the Kolmogorov-Smirnov test was used to check the adhesion of the residuals to the normal distribution curve. A significance level of *p*<0.05 was adopted for all analyses. A method of estimation was considered applicable for a determined group when the coefficient of determination (R^2^) was >0.7, which had the lowest RMSE among the methods evaluated, an ICC >0.7, and a CI of <95% with the smallest difference between the upper and lower limits.

The study was approved by the Ethics Research Committee of the Universidade Federal do Rio Grande do Norte (Federal University of Rio Grande do Norte), under approval number 308/2012 (CAAE 0290.0.051.000–11).

## Results

Of the total 14 nursing homes in Natal city, 4 refused to participate in the study, of which one was a non-profit organization and the other three were private institutions. Regarding the participants from the nursing homes that participated in the study, nine elderly people refused to participate, and eight were excluded because they did not meet the eligibility criteria; consequently, 168 elderly subjects living in 10 nursing homes participated in the study. The proportion of potencial lost participants was 35.9% of the total number of individuals eligible to participate in the study.

Most of those evaluated were women (76.8%). The average heights in female and male groups were 147.6 cm (7.0 cm) and 159.4 cm (9.0 cm), respectively. This difference (11.8 cm; p < 0,05) was statistically significant. A difference in the height measured was observed between the elderly people with respect to the age group; the 60–69 years group presented a mean height of 1.2 cm more than the 70–79 years group. Moreover, participants in this group were, on an average, 3.9 cm taller than the elderly participants aged up to 80 years. No significant differences were observed in other parameters with respect to age (Table 2).

**Table 2:** Characterization of the elderly population living in nursing homes according gender and age, Brazil, 2014.

	Total		Male		Female		60 to 69 years		70 to 79 years		Up to 80 years	
	n	μ (σ)	n	μ (σ)	n	μ (σ)	n	μ (σ)	n	μ (σ)	n	μ (σ)
**Age^a^^,^^b^**	168	79,9 (8,7)	39	76,0 (8,4)	129	81,1 (8,5)	23	65,5^c^ (2,5)	52	74,9^d^ (3,1)	93	86,3^e^ (4,9)
**Height^a^^,^^b^**	168	150,4 (9,0)	39	159,4 (9,0)	129	147,6 (7,0)	23	153,6^c^ (8,1)	52	152,4^c^^,^^d^ (9,6)	93	148,4^e^ (8,5)
**Knee height^a^**	168	47,7 (2,9)	39	50,3 (1,7)	129	46,9 (2,3)	23	47,9 (3,1)	52	48,1 (3,1)	93	47,4 (2,6)
**Ulna lenght^a^**	161	25,3 (1,7)	37	26,7 (5,7)	124	24,9 (1,5)	21	25,3 (1,9)	51	25,5 (1,8)	89	25,3 (1,6)
**Half arm span^a^**	151	77,2 (5,6)	36	81,7 (5,7)	115	75,8 (4,7)	18	78,8 (4,8)	49	77,5 (5,9)	84	76,6 (5,5)
**Demi span^a^**	151	70,0 (5,2)	36	73,8 (5,0)	115	68,8 (4,7)	18	71,3 (4,4)	49	70,2 (5,5)	84	69,5 (5,2)

a: Statistically significant *p-value* for t-test of mean difference between males and females;

b: Statistically significant p-value for ANOVA between age groups.

c,d,e: Bonferroni post-hoc test between age groups. Same letters indicate that there was no difference statistically significant between age. Different letters indicate that there was a statistically significant difference by the Bonferroni post-hoc test.

Qualitative analysis indicated that half-arm span and demi-span were difficult to measure due to the joint stiffness when the elderly were forced to maintain their arm in the correct position for the measurement. The ulna length and knee height measurements were more easily recorded, regardless of the physical condition of the elderly person.

**Fig 1:**
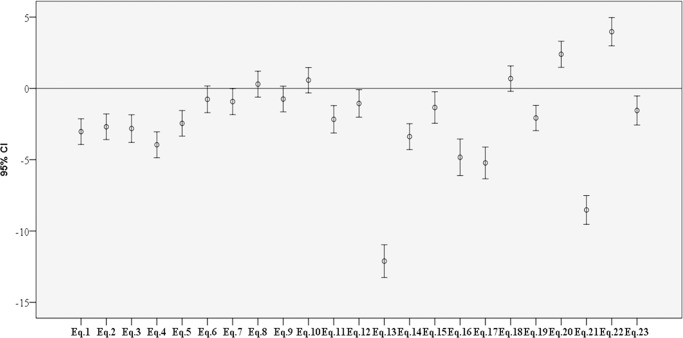
Bar chart of 95% confidence intervals of the mean difference between observed height and height estimated for elderly living in nursing homes (n = 168), Brazil, 2014.

**Fig 2:**
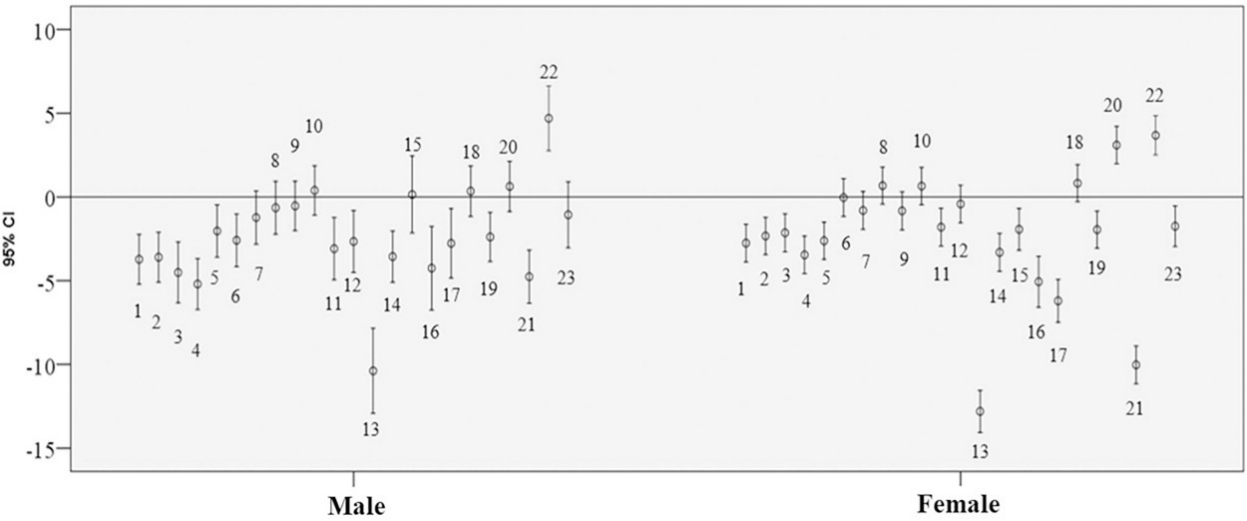
Bar chart of 95% confidence intervals of the mean difference between observed height and height estimated for elderly living in nursing homes stratified by sex, Brazil, 2014. (A) Numbers in the error bars correspond to the analyzed equations.

Analysis of the difference between the means of the estimation equations for the entire population studied revealed that only equations 8 and 10 by Bermúdez and Tucker and equation 18 by Lera et al. did not exhibit a statistically significant difference with measured height (Fig 1). In the analysis for male sex, only equations 7, 8, 9, and 10 by Bermúdez and Tucker, equation 15 by MAG, equations 18 and 20 by Lera et al., and equation 23 by Rabito et al. did not report a statistically significant difference between the male and female participants. For the female sex, equations 6, 7, 8, 9, and 10 by Bermúdez and Tucker, equation 12 by Palloni and Guend, and equation 18 by Lera et al. did not report a statistically significant difference (Fig 2).

In the analysis by age range, it was found that in the group of elderly persons aged 60–69 years, equations 3, 7–10, 11, 15, 18, 20, and 23 reported a difference of up to 3 cm and did not present a statistically significant difference with respect to mean height (Table 3). In the 70–79 years age group, no statistically significant difference was observed between the observed and estimated height for equations 7–10 by Bermúdez and Tucker, 18–20 by Lera et al., and 23 by Rabito et al. For the older elderly persons, no significant difference was observed between the mean observed and estimated heights using equation 6 by Chumlea et al., equations 8 and 10 by Bermúdez and Tucker, and equation 18 by Lera et al. (Table 3).

**Table 3:** Mean values and analysis of mean differences in stature and height estimated by predictive equations for elderly living in nursing homes stratified by age group, Brazil, 2014.

	60 to 69 years (n = 23)			70 to 79 years (n = 52)					Up to 80 years (n = 93)			
Method	μ (σ)	Diff. (σ)	95% CI Low-Upp	t	μ (σ)	Diff. (σ)	95% CI Low-Upp	t	μ (σ)	Diff. (σ)	95% CI Low-Upp	t
	153,6 (8,1)				152,4 (9,6)				148,4 (8,5)			
**Eq. 1**	154,2 (7,3)	4,3 (3,6)	2,7–5,8	5,7*	154,2 (7,3)	4,3 (5,4)	2,8–5,8	5,7*	154,2 (7,3)	3,4 (5,3)	2,3–4,5	6,2*
**Eq. 2**	153,8 (7,4)	3,1 (3,6)	1,5–4,6	4,1*	153,8 (7,4)	3,7 (5,4)	2,1–5,2	4,8*	153,8 (7,4)	3,4 (5,3)	2,3–4,5	6,2*
**Eq. 3**	153,9 (7,4)	1,5 (4,3)	-0,4–3,3	1,7	153,9 (7,4)	3,2 (6,0)	1,5–4,9	3,9*	153,9 (7,4)	4,3 (5,5)	3,2–5,4	7,5*
**Eq. 4**	155,1 (7,5)	5,3 (3,8)	3,7–7,0	6,7*	155,1 (7,5)	5,2 (5,4)	3,7–6,7	6,9*	155,1 (7,5)	4,3 (5,3)	3,2–5,4	7,8*
**Eq. 5**	153,8 (6,4)	3,5 (3,7)	1,9–5,1	4,5*	153,8 (6,4)	3,5 (5,5)	2,0–5,1	4,6*	153,8 (6,4)	3,4 (5,4)	2,3–4,6	6,2*
**Eq. 6**	151,8 (7,8)	2,7 (3,9)	1,1–4,4	3,4*	151,8 (7,8)	2,3 (5,5)	0,7–3,8	3,0*	151,8 (7,8)	0,7 (5,4)	-0,4–1,8	1,3
**Eq. 7**	152,1 (6,9)	-0,4 (3,8)	-2,1–1,2	-0,6	152,1 (6,9)	1,2 (5,7)	-0,4–2,8	1,6	152,1 (6,9)	2,7 (5,3)	1,6–3,8	4,8*
**Eq. 8**	150,8 (7,2)	-0,1 (3,8)	-1,8–1,5	-0,2	150,8 (7,2)	0,5 (5,6)	-1,0–2,1	0,7	150,8 (7,2)	0,6 (5,3)	-0,5–1,7	1,1
**Eq. 9**	151,9 (6,9)	-0,7 (3,7)	-2,3–0,9	-0,9	151,9 (6,9)	1,0 (5,6)	-0,6–2,5	1,2	151,9 (6,9)	2,5 (5,2)	1,4–3,6	4,6*
**Eq. 10**	150,6 (7,1)	-0,7 (3,6)	-2,3–0,8	-1,0	150,6 (7,1)	0,1 (5,5)	-1,4–1,7	0,2	150,6 (7,1)	0,5 (5,2)	-0,6–1,6	0,9
**Eq. 11**	153,8 (6,1)	1,4 (4,3)	-0,5–3,3	1,6	153,8 (6,1)	2,9 (5,8)	1,3–4,5	3,6*	153,8 (6,1)	4,2 (5,9)	3,0–5,4	6,9*
**Eq. 12**	152,6 (6,5)	2,3 (4,3)	0,4–4,1	2,5*	152,6 (6,5)	2,4 (5,8)	0,8–4,0	3,1*	152,6 (6,5)	2,1 (5,8)	0,9–3,3	3,4*
**Eq. 13**	163,3 (6,3)	11,5 (5,6)	8,9–14,0	9,4*	163,3 (6,3)	11,8 (7,2)	9,8–13,8	11,6*	163,3 (6,3)	13,8 (5,8)	12,5–15,0	22,5*
**Eq. 14**	155,1 (6,7)	1,7 (3,7)	0,1–3,3	2,1*	155,1 (6,7)	3,7 (5,7)	2,1–5,3	4,6*	155,1 (6,7)	4,9 (5,3)	3,8–6,0	8,7*
**Eq. 15**	154,3 (6,0)	0,0 (3,8)	-2,1–2,0	0,0	154,3 (6,0)	0,4 (6,1)	-1,5–2,4	0,4	154,3 (6,0)	2,4 (6,4)	0,8–4,0	3,0*
**Eq. 16**	154,3 (11,2)	3,0 (5,4)	0,3–5,7	2,3*	154,3 (11,2)	2,4 (8,0)	0,1–4,7	2,1*	154,3 (11,2)	4,2 (7,4)	2,6–5,8	5,2*
**Eq. 17**	155,7 (8,2)	3,4 (4,2)	1,4–5,5	3,5*	155,7 (8,2)	3,5 (6,5)	1,6–5,3	3,8*	155,7 (8,2)	5,8 (5,9)	4,5–7,1	9,0*
**Eq. 18**	150,5 (6,9)	-0,6 (3,7)	-2,2–1,0	-0,8	150,5 (6,9)	0,1 (5,5)	-1,4–1,6	0,1	150,5 (6,9)	0,4 (5,3)	-0,7–1,5	0,7
**Eq. 19**	153,3 (6,9)	2,2 (3,8)	0,6–3,9	2,8*	153,3 (6,9)	2,8 (5,4)	1,3–4,4	3,8*	153,3 (6,9)	3,2 (5,3)	2,1–4,3	5,9*
**Eq. 20**	148,7 (7,7)	-1,5 (3,8)	-3,1–0,1	-1,9	148,7 (7,7)	-1,2 (5,5)	-2,8–0,3	-1,6	148,7 (7,7)	-2,0 (5,4)	-3,1 –-0,9	-3,6*
**Eq. 21**	160,1 (5,2)	6,9 (3,8)	5,2–8,5	8,7*	160,1 (5,2)	8,5 (6,3)	6,8–10,3	9,8*	160,1 (5,2)	11,1 (5,7)	10,0–12,3	18,9*
**Eq. 22**	146,8 (7,6)	-4,5 (3,5)	-6,2 –-2,8	-5,5*	146,8 (7,6)	-4,9 (5,9)	-6,6 –-3,2	-5,8*	146,8 (7,6)	-3,8 (5,4)	-5,0 –-2,6	-6,4*
**Eq. 23**	152,1 (8,1)	1,1 (3,5)	-0,7–2,8	1,3	152,1 (8,1)	0,6 (6,1)	-1,2–2,3	0,6	152,1 (8,1)	1,6 (5,6)	0,4–2,8	2,6*

*: Value p <0.05 by Student's t-test for repeated samples. μ: mean; σ: standard deviation; Diff: mean difference between observed and estimated height; CI (95%): 95% confidence interval, upper and lower; T: value of t-Student test.

**Fig 3:**
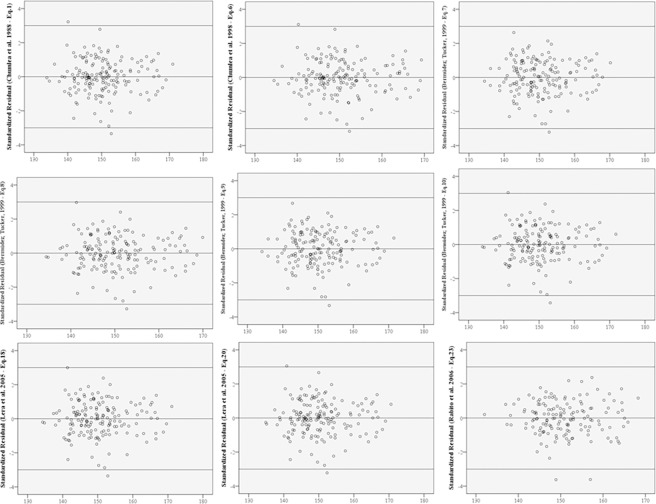
Bland-Altman plot of the height-estimation equations, Brazil, 2014.

Concordance analysis found that equation 10 by Bermúdez and Tucker could be applied for the entire study population, elderly men, and those aged between 60 and 69 years (Tables 4 and 5). None of the equations were applicable for elderly women or individuals >70 years. Residual normality was observed for all the equations analyzed. In the Bland-Altman plots, it was observed that in all the graphs, the points displayed random dispersion around the zero line, resulting in a uniform cloud. The residuals are, therefore, independent, with a zero mean and uniform variance, satisfying the assumptions of linear regression analysis (Fig 3).

**Table 4:** Analysis of concordance of observed and estimated height of elderly living in nursing homes and stratified by sex, Brazil, 2014.

	Total				Male				Female			
Method	R^2^	RMSE	ICC	95% CI Low-Upp	R^2^	RMSE	ICC	95% CI Low-Upp	R^2^	RMSE	ICC	95% CI Low-Upp
**Eq. 1**	0,676	5,151	0,725	0,375–0,858	0,792	4,146	0,718	0,168–0,888	0,428	5,338	0,512	0,188–0,700
**Eq. 2**	0,677	5,136	0,742	0,456–0,860	0,787	4,204	0,725	0,199–0,889	0,434	5,307	0,533	0,257–0,701
**Eq. 3**	0,616	5,602	0,704	0,429–0,830	0,787	4,204	0,522	0,031–0,771	0,391	5,507	0,506	0,261–0,669
**Eq. 4**	0,671	5,179	0,694	0,197–0,858	0,785	4,218	0,638	-0,039–0,866	0,433	5,317	0,479	0,085–0,698
**Eq. 5**	0,681	5,102	0,708	0,413–0,837	0,783	4,233	0,759	0,508–0,879	0,434	5,311	0,470	0,152–0,667
**Eq. 6**	**0,658**	**5,281**	**0,791**	**0,716–0,846**	0,778	4,293	0,736	0,405–0,875	0,424	5,357	0,601	0,478–0,700
**Eq. 7**	0,650	5,343	0,760	0,664–0,827	0,787	4,204	0,761	0,575–0,870	0,391	5,507	0,545	0,396–0,662
**Eq. 8**	**0,666**	**5,216**	**0,796**	**0,733–0,845**	0,794	4,135	0,779	0,617–0,877	0,432	5,323	0,601	0,478–0,701
**Eq. 9**	0,661	5,267	0,770	0,686–0,832	**0,787**	**4,204**	**0,830**	**0,699–0,907**	0,391	5,507	0,554	0,408–0,669
**Eq. 10**	**0,681**	**5,111**	**0,802**	**0,740–0,850**	**0,787**	**4,193**	**0,838**	**0,712–0,912**	0,430	5,325	0,606	0,484–0,705
**Eq. 11**	0,607	5,663	0,658	0,406–0,791	0,787	4,204	0,559	0,197–0,766	0,391	5,507	0,366	0,138–0,543
**Eq. 12**	0,616	5,596	0,719	0,595–0,803	0,781	4,254	0,590	0,264–0,779	0,429	5,335	0,457	0,297–0,589
**Eq. 13**	0,493	6,321	0,278	-0,083–0,614	0,338	7,567	0,257	-0,101–0,584	0,298	5,762	0,145	-0,066–0,419
**Eq. 14**	0,621	5,346	0,670	0,286–0,827	0,729	4,263	0,678	0,181–0,862	0,310	5,578	0,396	0,081–0,608
**Eq. 15**	0,457	6,090	0,635	0,512–0,732	0,333	6,646	0,540	0,247–0,741	0,185	5,554	0,384	0,187–0,551
**Eq. 16**	0,561	5,812	0,688	0,520–0,792	0,581	6,010	0,699	0,421–0,846	0,396	5,218	0,550	0,360–0,686
**Eq. 17**	0,561	5,812	0,645	0,239–0,815	0,581	6,010	0,724	0,484–0,856	0,396	5,218	0,481	0,040–0,715
**Eq. 18**	**0,677**	**5,133**	**0,796**	**0,733–0,846**	**0,794**	**4,137**	**0,821**	**0,683–0,902**	0,429	5,550	0,593	0,468–0,694
**Eq. 19**	0,681	5,105	0,746	0,531–0,850	0,792	4,145	0,786	0,487–0,901	0,429	5,336	0,514	0,270–0,675
**Eq. 20**	**0,669**	**5,197**	**0,793**	**0,710–0,851**	**0,794**	**4,134**	**0,824**	**0,689–0,904**	0,434	5,309	0,571	0,401–0,695
**Eq. 21**	0,619	5,570	0,365	-0,098–0,687	0,787	4,204	0,624	0,004–0,850	0,391	5,507	0,198	-0,080–0,503
**Eq. 22**	0,624	5,345	0,691	0,292–0,843	0,615	5,850	0,662	0,228–0,846	0,416	5,074	0,522	0,131–0,729
**Eq. 23**	**0,615**	**5,445**	**0,775**	**0,700–0,833**	0,594	5,994	0,749	0,564–0,863	0,411	5,150	0,631	0,506–0,730

R^2^: coefficient of determination; RMSE: root mean square error; ICC: intraclass correlation coefficient; 95% CI: 95% confidence interval, upper and lower.

**Table 5:** Analysis of concordance of observed and estimated height of elderly living in nursing homes stratified by age group, Brazil, 2014.

	60 to 69 years (n = 23)				70 to 79 years (n = 52)				Up to 80 years (n = 93)			
Method	R^2^	RMSE	ICC	95% CI Low-Upp	R^2^	RMSE	ICC	95% CI Low-Upp	R^2^	RMSE	ICC	95% CI Low-Upp
**Eq. 1**	0,805	3,663	0,768	0,050–0,928	0,684	5,434	0,717	0,291–0,871	0,608	5,350	0,681	0,382–0,823
**Eq. 2**	0,803	3,680	0,832	0,390–0,942	0,677	5,493	0,745	0,426–0,875	0,612	5,323	0,688	0,391–0,827
**Eq. 3**	0,755	4,094	0,859	0,695–0,938	0,612	6,026	0,729	0,490–0,852	0,575	5,571	0,628	0,224–0,806
**Eq. 4**	0,787	3,820	0,729	-0,044–0,919	0,677	5,495	0,687	0,137–0,868	0,612	5,328	0,646	0,221–0,822
**Eq. 5**	0,790	3,787	0,795	0,252–0,930	0,686	5,420	0,715	0,407–0,854	0,626	5,231	0,639	0,336–0,793
**Eq. 6**	0,781	3,879	0,838	0,507–0,939	0,672	5,528	0,783	0,621–0,876	0,596	5,434	0,745	0,640–0,823
**Eq. 7**	**0,789**	**3,808**	**0,891**	**0,762–0,952**	0,651	5,712	0,783	0,651–0,869	0,615	5,309	0,692	0,489–0,809
**Eq. 8**	**0,789**	**3,806**	**0,892**	**0,762–0,953**	0,659	5,639	0,796	0,670–0,878	0,610	5,336	0,744	0,638–0,823
**Eq. 9**	**0,799**	**3,710**	**0,895**	**0,771–0,954**	0,661	5,624	0,794	0,668–0,876	0,627	5,218	0,706	0,518–0,816
**Eq. 10**	**0,806**	**3,653**	**0,895**	**0,771–0,954**	0,672	5,528	0,803	0,680–0,882	0,624	5,239	0,754	0,651–0,830
**Eq. 11**	0,717	4,402	0,834	0,650–0,926	0,635	5,835	0,717	0,495–0,841	0,546	5,755	0,547	0,194–0,739
**Eq. 12**	0,716	4,421	0,810	0,559–0,919	0,645	5,765	0,736	0,549–0,847	0,539	5,807	0,637	0,479–0,751
**Eq. 13**	0,567	5,527	0,371	-0,086–0,747	0,420	7,305	0,299	-0,100–0,632	0,527	5,801	0,242	-0,068–0,585
**Eq. 14**	0,785	3,383	0,863	0,679–0,942	0,638	5,730	0,715	0,398–0,856	0,575	5,326	0,573	0,079–0,789
**Eq. 15**	0,719	3,981	0,843	0,604–0,942	0,543	6,203	0,713	0,518–0,837	0,323	6,348	0,511	0,295–0,675
**Eq. 16**	0,689	4,150	0,758	0,426–0,905	0,546	6,251	0,702	0,523–0,821	0,548	5,676	0,656	0,404–0,796
**Eq. 17**	0,689	4,150	0,750	0,245–0,913	0,546	6,251	0,689	0,429–0,830	0,548	5,676	0,593	0,060–0,809
**Eq. 18**	**0,794**	**3,768**	**0,891**	**0,763–0,952**	0,672	5,536	0,800	0,675–0,880	0,624	5,239	0,743	0,636–0,822
**Eq. 19**	0,787	3,834	0,857	0,627–0,942	0,682	5,454	0,764	0,547–0,873	0,630	5,197	0,675	0,399–0,814
**Eq. 20**	**0,789**	**3,817**	**0,876**	**0,724–0,946**	0,676	5,504	0,807	0,686–0,884	0,602	5,389	0,730	0,593–0,822
**Eq. 21**	0,819	3,520	0,575	-0,091–0,865	0,602	6,100	0,434	-0,095–0,740	0,599	5,413	0,288	-0,087–0,628
**Eq. 22**	0,776	3,518	0,738	-0,020–0,925	0,598	5,891	0,666	0,207–0,846	0,587	5,372	0,678	0,329–0,831
**Eq. 23**	0,774	3,527	0,877	0,706–0,952	0,587	5,964	0,767	0,621–0,861	0,576	5,488	0,744	0,624–0,828

R^2^: coefficient of determination; RMSE: root mean square error; ICC: intraclass correlation coefficient; 95% CI: 95% confidence interval, upper and lower.

## Discussion

The present census-type study employed a rigorous methodology for the comparison and validation of height estimating equations among elderly persons living in nursing homes. Among the studies that aim to evaluate methods for estimating the height in the elderly, this study was different since several statistical tests and their indices, such as the ICC and the corresponding 95% CI, were used. Meanwhile, most of the studies are restricted to using the Pearson correlations, Student’s t-test for paired samples, or Bland-Altman plot [21–29]. This pioneering concept, however, has led to difficulties in finding studies with a similar objective and methodology to compare the results and discuss them.

From the results observed, it was found that equation 10 by Bermudéz and Tucker was the most applicable for elderly men and individuals aged between 60 and 69 years. In the other groups (elderly and those aged 70 years or older), none of the methods were applicable for estimating height. The estimation of height is therefore not recommended for these groups.

The proper assessment of height is essential, as it is the basic measurement for precise indicators of nutritional status assessment, such as BMI. The use of a non-applicable estimation equation can lead to errors in nutritional status indicators. Stature combined with weight can be used to calculate creatinine height index, basal energy expenditure, basal metabolic rate, vital capacity, estimation of nutrient requirements, and calculation of body composition[22].

In clinical practice, such errors can result in inadequate dietetic and/or drug interventions. In turn, these inadequacies can increase nutritional risk and morbidity and mortality. In public health practice or in epidemiological studies, estimates from equations that are not validated for the studied population can lead to inadequate assessments of the population profile in question and misleading results[21,30].

To minimize these errors, an applicable estimation method should be chosen. When no validated equation for the population concerned is available, it is preferable not to estimate height by questionable methods. Thus, the use of other indicators such as perimeter and/or skinfold measurements provides more reliable information about nutritional status.

While equation 10 by Bermúdez and Tucker was the most applicable for elderly men, none of the equations met the applicability requirements for elderly women. In terms of age group, it was noted that equation 10 by Bermúdez and Tucker was the most applicable for elderly persons aged 60–69 years. This equation was also the most applicable for subsequent age groups, although there was a loss of precision with increasing age. As a result, the use of these equations in the age groups 70–79 years and 80 years or more is not recommended. None of the methods of estimation evaluated were applicable for institutionalized elderly women or elderly persons aged 70 or more.

Following analysis of the means of the estimating equations, it was found that only equations 8 and 10 by Bermúdez and Tucker, 18 by Lera et al., and 23 by Rabito et al. did not exhibit a statistically significant difference between measured and estimated height. Rabito et al. found a statistically significant difference between the observed and estimated height obtained using equation 1 by Chumlea et al. Sampaio et al.[31] found that there was a statistically significant difference of 3.5 cm between the observed and estimated height (35 elderly men and 29 elderly women) obtained using equation 1 by Chumlea et al. Fogal et al. [27] found that the equation of Chumlea et al. [8] was not adequate to estimate the height of the study sample, especially for elderly women. These results corroborate with those of the present study.

Stratified analysis by age group revealed an increase in the mean differences with increasing age. Younger elderly persons had greater mobility and less curvature of the spine [32], resulting in an easier measurement of the observed height and greater accuracy of the equations in this age group. Therefore, the methods of estimating height for elderly should consider the reduced mobility of the elderly as age increases. An effective estimation method should be applicable to oldest elderly persons, as such methods are especially likely to be applied to this group. It is noteworthy that all the equations where there was no statistically significant difference for the group aged 80 or older were specifically developed for the elderly.

Concordance analysis stratified by sex found that equation 10 by Bermúdez and Tucker exhibited greater applicability for men, with a more precise CI. None of the equations were considered applicable for female elderly persons. The use of the equations analyzed to estimate the height of elderly women is therefore not recommended.

Regarding the analysis of concordance by age, it was found that equations 9 and 10 by Bermudez and Tucker were the most applicable for the elderly persons studied. However, none of the methods analyzed could be recommended for assessing the height among those aged 70 years or more.

The results for women and older elderly persons behaved similarly. The RMSE of both strata was greater than for the other groups, and there was a reduction in the coefficients of determination, ICC, and CI precision. This result was explained by the fact that 86% of the elderly persons aged 80 and over were women. As the two groups contained the same individuals, they presented similar results.

The equations by Rabito et al.[15] and Silveira et al.[17] were used in the present study as these articles are based on Brazilian and hospitalized individuals, with the latter being similar to institutionalized elderly. When choosing an estimating equation for a certain individual, considering factors related to both the evaluated and the evaluator is necessary. For the evaluation, sex and age should be considered, while it is necessary that the evaluator is trained in the collection of anthropometric measurements and should possess calibrated equipment for the optimal measurement of the anthropometric site to be measured[19].

One possible limitation of this study was the lack of a gold standard anthropometrist for the calibration of the evaluators. To minimize errors, however, the evaluators were trained and calibrated using TEM. Only those with errors similar to those of an experienced anthropometrist were considered suitable for field work. Another limiting aspect of the study was the difficulty experienced in anthropometric data collection, as the stiffness observed in the elderly resulted in difficulty in half arm spam and demi measurements. Moreover, difficulty was encountered in maintaining the correct position for the time required to perform the measurement in duplicate. To overcome this problem, an assistant supported the arm of the elderly person being assessed, maintaining a 90° position. While the elderly people with mobility restrictions were frequently not optimally positioned for anthropometric assessment, knee height and ulna length required smaller movements in bed and were therefore easier to apply.

It is recommended, therefore, that estimations of the height of institutionalized elderly persons is performed in accordance with the results observed. When a valid method cannot be used, the use of other nutritional status indicators is recommended, such as perimeters and/or skinfolds, which provide more reliable information than a questionable estimate.
